# Noise Annoyance and Sleep Disturbance Due to Road Traffic and Railway Noise in Germany

**DOI:** 10.3390/ijerph22091366

**Published:** 2025-08-30

**Authors:** Sarah Leona Benz, Julia Kuhlmann, Jonas Bilik, Manfred Liepert, Dirk Schreckenberg

**Affiliations:** 1 ZEUS GmbH, Centre for Applied Psychology, Environmental and Social Research, 58093 Hagen, Germany; bilik@zeusgmbh.de (J.B.); schreckenberg@zeusgmbh.de (D.S.); 2 Möhler + Partner Ingenieure GmbH, 86153 Augsburg, Germany; manfred.liepert@mopa.de

**Keywords:** noise annoyance, sleep disturbance, transportation noise, exposure–response assessment, urbanisation

## Abstract

Environmental noise exposure is omnipresent, but the type of noise source and its appraisal may differ in varying contexts. For instance, studies have found significant differences in annoyance ratings between urbanisation levels. In this article, a re-analysis of existing survey data is presented, assessing noise annoyance and sleep disturbance from road traffic and railway noise in a random sample stratified by rural, suburban, and inner-city areas. Noise exposure was estimated using modelled *L*_den_ and *L*_night_ levels. Exposure–response curves showed greater annoyance at lower road traffic noise levels compared to the WHO guidelines (10% highly annoyed at 35 dB *L*_den_ vs. WHO 53 dB *L*_den_). Railway noise annoyance aligned with the WHO estimates; however, sleep disturbance was lower at comparable exposure levels (3% highly sleep-disturbed at 53 dB *L*_night_ vs. WHO 44 dB). This re-analysis provides robust exposure–response relationships. The findings indicate higher levels for road traffic noise annoyance in Germany compared to international standards. A resulting policy implication is to link regular population surveys to noise action planning as a form of public participation. This approach enables the development of measures tailored to local conditions and supports the estimation of potential impacts, such as the number of people who may benefit from reduced noise exposure.

## 1. Introduction

Environmental noise exposure is an environmental stressor and can elicit psychological responses including noise annoyance and physiological stress reactions. Long-term exposure to environmental noise can lead to chronic stress, ultimately increasing the risk for various adverse health effects. The main effects of environmental noise are sleep disturbance and annoyance, primarily linked to road traffic noise [1].

In 2018, the WHO published the Environmental Noise Guidelines for the European Region, recommending—based on systematic reviews and meta-analyses including international studies (e.g., [2,3])—exposure levels for different environmental noise sources above which adverse health effects are to be expected: 10% highly annoyed people and 3% highly sleep-disturbed people [4]. Based on exposure–response functions that depict the relation between percentage highly annoyed (%HA) and exposure levels, 10%HA is reached at 53 dB (throughout this paper, we refer to A-weighted sound level metrics in decibels as defined in the EU Directive 2002/49/EC [5] for the quantification of noise exposure and use the notation dB in line with ISO 1996-1:2016 [6] and the SI principles [7]) *L*_den_ for road traffic and at 54 dB *L*_den_ for railway noise [4]. Regarding annoyance due to road traffic and railway noise, the authors found 2.7 and 3.4 higher odds of being highly annoyed per 10 dB *L*_den_ increase, respectively. Similar estimates were found in the LIFE adult study [8]: the risk of being highly annoyed was 3.5-fold for road traffic noise and 3.3-fold for railway noise per 10 dB *L*_den_ increase, although the prevalence of %HA was slightly lower for road traffic and railway noise in comparison to the WHO curves. It is noteworthy that the noise exposure modelling included tram noise and secondary road networks, allowing for a more accurate depiction of the actual noise exposure levels compared to the available exposure data analysed in the WHO curves. In 2011, the World Health Organization (WHO) estimated that approximately 900,000 disability-adjusted life years (DALYs) are lost annually within the EU’s urban population due to sleep disturbance related to environmental noise and more than 650,000 DALYs due to noise annoyance [1].

Exposure–response functions can be instrumental in noise monitoring and can provide—in combination with the available population data within a study area—a foundation for estimating the absolute number of individuals adversely affected by environmental noise. For instance, noise mapping under the EU Environmental Noise Directive encourages prioritising quantifications of health impacts attributable to environmental noise. To achieve this, the generalised exposure–response functions specified in Annex III of the Directive, which are derived from the WHO reviews, should be consulted.

**Figure 1:**
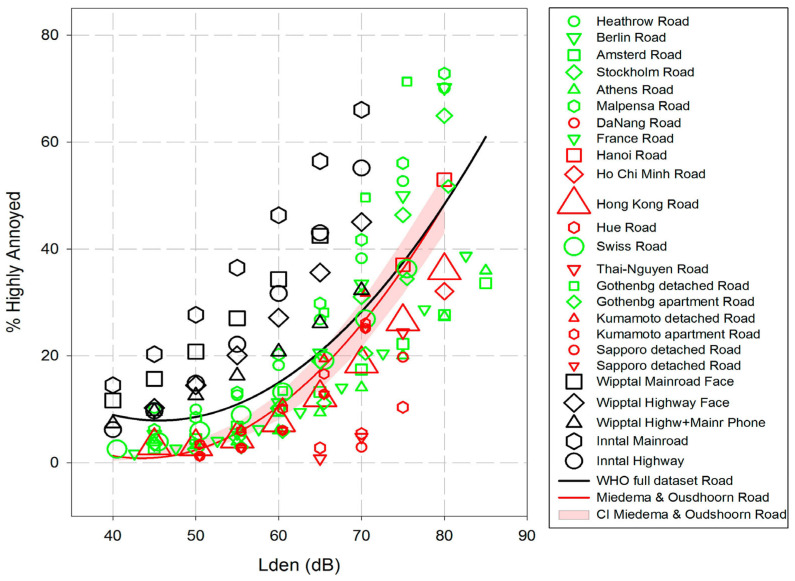
Exposure–response relationships for %HA with *L*_den_ for road traffic noise of single studies used in the WHO’s meta-analysis [2].

Further, exposure–response relationships can be used to estimate the effectiveness of noise abatement measures. These estimations depend on the robustness of the exposure–response functions and how well they reflect the local (regional, national) noise impact conditions. The advantage of generalised exposure–response functions, such as those derived in the WHO reviews, is that they are based on the results of multiple international studies and thus appear to be robust and generally applicable. However, the WHO review on annoyance due to environmental noise also illustrates how heterogeneous the exposure–response relationships from individual original studies can be (see Figure 1, taken from [2]).

For regional or national noise abatement planning, such as in the context of noise action planning under the EU Environmental Noise Directive, regionally or nationally valid exposure–response functions for quantifying the health effects of environmental noise would be preferable.

Differences in noise–exposure relationships can be the results of numerous factors, e.g., differences in attitudes towards a noise source, different typologies, or the level of urbanisation of study areas.

People living in larger cities are especially prone to being exposed to a variety of environmental factors. Aligning with the legislative endeavour to promote the “city of short distances” accompanied by a redensification of cities, housing and emitting sources are located in close proximity to each other. Residing in urban environments is often associated with exposure to distinct noise sources compared to rural settings. For instance, people living in urban areas typically experience more traffic and commercial noise, whereas individuals in rural regions may be more frequently exposed to wind turbine noise or noise from agricultural equipment. However, not only acoustic aspects may differ (e.g., type of noise source); non-acoustic factors can play a role as well: The relevance of the same noise source can vary across diverse environments. In rural areas, for example, there can be greater expectations of a quiet environment [9].

In 2021, 6600 people participated in the Canadian Perspectives on Environmental Noise Survey that assessed noise annoyance caused by transportation (road, trains, and aircraft/helicopter) and construction noise considering three geographic areas, i.e., urban, suburban, and remote/rural areas [9]. Noise annoyance was measured in line with ISO/TS 15666 with the addition of a “do not hear” answer option [10], while noise exposure levels were not assessed. The odds of being highly annoyed were significantly higher for urban areas compared to remote/rural areas for almost all noise sources, except for railway noise. In multivariate logistic regression models, in which variables such as noise sensitivity and perception of the living area as quiet, calm, and relaxing were considered as well, significant results can only be found for the odds of being highly annoyed and construction noise. Nevertheless, the authors argue that geographic regions need to be considered in noise impact studies.

The German Health Update (GEDA) study considered three district types when assessing noise annoyance due to road and air traffic, railway, and neighbours: independent metropolitan cities with ≥100,000 inhabitants, urban districts, and rural districts [11]. People living in metropolitan areas reported higher noise annoyance compared to people living in urban and rural districts. However, as no noise exposure levels were assessed, it is unclear whether this difference might be due to differences in noise exposure.

Considering noise exposure, the NORAH (Noise-Related Annoyance, Cognition, and Health) study was one of the most extensive noise impact studies conducted in Germany [12]. The aim of the NORAH study was to assess the impact of traffic noise, with a focus on aircraft noise, on residents’ health, quality of life, and children’s cognitive development. The study areas were regions around major German airports: Frankfurt, Cologne-Bonn, and Berlin. Both noise impacts and exposure levels were assessed. The study found that in the vicinity of Frankfurt Airport, aircraft noise annoyance for the same aircraft noise levels was higher in quieter areas (i.e., low background noise) than in otherwise “louder” areas [13].

To derive nationally valid and robust exposure–response functions for road traffic and railway noise, we used existing data from the most recently conducted socio-acoustic survey commissioned by the German Environment Agency [14], which includes noise annoyance and sleep disturbance ratings as well as noise exposure assessments. In addition, the data allowed for potential differences in noise annoyance ratings between urbanisation levels to be identified.

Given the interplay of acoustic and non-acoustic factors across different geographic settings, it was hypothesised that noise annoyance and sleep disturbance vary systematically with the degree of urbanisation. Specifically, individuals living in rural or less urbanised areas may report higher levels of annoyance and sleep disturbance at comparable noise exposure levels, potentially due to greater expectations of environmental quiet, lower baseline noise levels, and differing attitudes toward specific noise sources.

## 2. Materials and Methods

### 2.1. Procedure

Data from a survey study on the environmental noise annoyance situation in Germany conducted by the authors were used for re-analysis. The survey study was conducted in two survey waves, the first in autumn 2018 and the second in spring 2019. In the initial survey wave, a random sample of 18,028 households received a postal invitation letter. This letter included a cover letter from the project team introducing the survey’s purpose—to assess and improve our understanding of noise exposure impacts on humans and their living conditions. Additionally, a QR code for participation in the online survey, a data protection declaration, and a letter of reference from the German Environment Agency were provided. Due to a low response rate in the first survey wave, a second sample of 12,000 were invited stepwise in spring 2019 to participate in the survey. In addition to the listed study documents from survey wave 1, they received a printed questionnaire and a stamped return envelope. The average time required to complete the survey was around 20 min.

### 2.2. Selection of Study Areas

To consider potential varying degrees of noise exposure and exposure to different noise sources depending on the region and population density, four representative areas in Northern, Southern, Eastern, and Western Germany were selected, each divided into three levels of urbanisation (inner-city, suburban, and rural): Hamburg (North), Dresden (East), Stuttgart (South), and Dusseldorf (West). They fulfilled the following criteria: metropolitan area, presence of an airport, availability of comprehensive EU noise mapping data for road, rail, and air traffic, the distance between the metropolitan areas, and coverage of all four geographical areas of Germany (North, East, South, and West). Noise exposure levels were not considered for the selection of study areas. National statistics of the population density and estimations of the population per km^2^ were used to define the level of urbanisation [15]. For the three categories of level of urbanisation, the number of participants was based on the ratio of the population density of each area. For all study areas except Dusseldorf, a ratio of 4:2:1 for inner-city/suburban/rural area was applied. In the less rural area of Dusseldorf, the ratio slightly differed with 6:3:1.

### 2.3. Sample Selection and Participation

To estimate the required sample size for the analyses, power analyses were performed with G-Power 3.1.2 using odds ratios (ORs) for noise annoyance due to the different sources provided in [2]. The smallest OR identified was 2.78 for noise annoyance due to road traffic noise. A conservative sample size estimation for the initially planned analyses revealed a sample size of 2000 participants. For the logistic regression analyses conducted in this paper, a sample size of 912 participants was found to be sufficient. 

First, 20,000 buildings within the study areas were randomly selected in line with the region-specific distribution key for the first survey wave for which personal data of the adult residents (first name, last name) were provided by an address service company. A random sample of 20,000 people living in these 20,000 buildings was drawn based on the region-specific distribution key (response rate estimation of 10%). As there were not sufficient data for some of the area types, 18,028 people were contacted via mail in the first survey wave.

A similar sampling procedure was applied for the second survey wave in May 2019: 24,000 buildings were randomly selected based on the region-specific distribution key. The address service company supplied personal data on the adult population and a random sample of 12,000 people was drawn. Then, the sample was split in two: The first 6000 people were contacted and invited to participate in May 2019. Two weeks later, the second half of the sample received the invitation to participate, while the first 6000 people were sent a reminder.

Complete participation and data are available for 749 people for the first survey wave and for 1251 people for the second survey wave. The sample size of 1971 translates into a total response rate of 7.1% with 4.7% for the first (online only) and 10.3% for the second survey wave (paper–pencil and online; calculation based on [16]). Recent surveys conducted within noise impact studies attain response rates up to 31% [17], depending on the type of noise studied and method of data collection. The unexpectedly low response rate of 4.7% in the first survey wave could be attributed to the mode switch people were asked to undertake: receiving a survey in the mail but needing to participate online. In addition, people with limited or no digital literacy may have been excluded from participation due to the online format of the initial survey wave. When people received the questionnaire by mail and were given the option between two modes of participation (online and postal) in the second survey wave, the response rate improved and fell within the expected range with 10.3%.

For the purpose of this paper, we categorised the original sample into four subgroups: people who were exposed to (1) ≥35 dB *L*_den_ for road traffic (N = 900), (2) ≥35 dB *L*_night_ for road traffic (N = 730), (3) ≥35 dB *L*_den_ for railway (N = 1099), and (4) ≥35 dB *L*_night_ for railway (N = 753).

### 2.4. Noise Exposure Assessment for Road and Rail

To determine the sample households’ address-related exposure levels to the different noise sources, input data from the noise mapping (including buildings and rail), the results of the noise mapping, both based on 2017 EU mapping, and a digital terrain model were acquired from responsible state authorities. Models were developed based on these input data using SoundPlan 8.0 to calculate the exposure levels for the survey years. The use of input data from the EU noise mapping allowed only for the modelling of noise exposure to major roads; less busy roads were not included. The A-weighted noise metrics *L*_den_ and *L*_night_ were modelled for road and rail noise with the 24 h value *L*_den_, including day (6 am to 6 pm), evening (6 pm to 10 pm), and night (10 pm to 6 am). A weighting penalty of 5 dB and 10 dB was applied for evening and night, respectively. For a more detailed description of the noise level assessment, see [14].

### 2.5. Questionnaire

The questionnaire contained items on residential conditions, annoyance due to different noise sources (e.g., road, rail), sleep disturbances due to those noise sources, personal factors, and socio-demographics. Items from previous socio-acoustic surveys served as a basis for the questionnaire (e.g., residential conditions [12,18,19]). Residential satisfaction was evaluated with the question, ‘How satisfied are you with your living environment?’. The participants rated their satisfaction on a five-point scale from (1) ‘not satisfied’ to (5) ‘very satisfied’. Research has shown that residential satisfaction is linked to noise annoyance [20], particularly when the focus is on the broader residential area rather than the dwelling itself [21]. A similar item with an identical five-point scale was used to assess the participants’ satisfaction with their own flat or house (‘How satisfied are you with your flat/house?’). The health status of the participants was measured with a single item and answered on a five-point scale ranging from (1) ‘excellent’ to (5) ‘bad’: when you think about the last 4 weeks, how would you describe your health status in general?

The key indicators for effects from noise exposure in this study were the percentage of highly annoyed people (%HA) and the percentage of highly sleep-disturbed people (%HSD). Both of these indicators are used in the European noise action planning [5] as well as in the WHO Noise Guidelines [4]. Noise annoyance was assessed using the standardised question ‘Thinking about the last 12 months, when you are here at home, to what extent are you personally bothered, annoyed or disturbed by noise from <noise source>?’ to be answered on a 5-point verbal scale with the verbal descriptors ‘not at all’, ‘slightly’, ‘moderately’, ‘very’, and ‘extremely’ following the recommendations of the International Commission on Biological Effects of Noise (ICBEN) [10,22,23]. Similarly, self-reported sleep disturbance attributable to specific noise sources (road and rail) was assessed separately with three items for the sleep stages of falling asleep, sleeping through the night, and sleeping in, anchored to the question ‘Thinking about the last twelve months at your home, during night-time when you want to sleep, how much did <noise source> noise bother, disturb, or annoy you?’, using the same 5-point verbal scale [10,22,23]. An average sleep disturbance score was calculated from the answers to these three individual questions. Participants selecting either of the two highest noise annoyance response categories (‘very’ (4) or ‘extremely’ (5)), corresponding to the upper 40% of the rating scale, were classified as ‘highly annoyed’ (HA_V_). The same cut-off (upper 40% of the scale) was used for highly sleep-disturbed people, which translates into a cut-off score of 3.67 on the sleep disturbance scale averaged over three items. The index v hints to the use of the verbal 5-point scale as the basis for the HA and HSD definition according to the ISO/TS 15,666 nomenclature. Noise annoyance due to noise from road and railways and vehicle-specific noise sources (e.g., separate assessment of annoyance from road noise sources for cars, trucks, motorcycles, and motorways) was assessed.

Research shows that several non-acoustic factors have an influence on noise annoyance and sleep disturbance [24,25,26,27,28]. Noise sensitivity is regarded as a stable personality trait that reflects an individual’s overall susceptibility to noise [29], and it has been shown to alter the exposure–response relationship of noise health effects (e.g., [30,31,32]). It was assessed using a time-efficient single item ‘How sensitive are you to noise in general?’ to be answered on a five-point scale from (1) not sensitive to (5) very sensitive [33]. Socioeconomic status was operationalised using the Scheuch-Winkler-Index (SWI, [34,35]), which was composed of the items highest school degree, vocational training, occupational position, and income. The SWI lies between 3 and 21. Three categories of low, medium, and high for the SWI were formed.

### 2.6. Ethical Considerations

Given the survey mode of the study, which relies on self-reported data from participants, no ethical voting or institutional review board approval was required. Participants were informed about the nature of the research, ensuring their voluntary participation and confidentiality of their responses. The data were stored in a secure environment and used for scientific purposes only.

### 2.7. Statistical Analyses

Descriptives including the mean, standard deviation, median, and frequencies were calculated for the four subgroups and for the four subgroups divided into the three different levels of urbanisation. The variable for health status was recoded for a high rating to reflect good health and a lower rating to indicate a worse self-reported health status. Histograms were created to display the frequency distribution of noise exposure. Correlational analyses using Pearson’s *r* were performed to assess the relationship between noise annoyance due to different vehicle-specific noise exposures (e.g., cars, trucks, motorcycles) and noise annoyance rated for main noise sources (road, rail). In addition, correlations were calculated between the exposure levels (*L*_den_ and *L*_night_, respectively) and the noise annoyance ratings as well as the self-reported sleep disturbance ratings and non-acoustical factors (satisfaction with neighbourhood, satisfaction with living environment, health status, age, noise sensitivity, and the SWI).

To assess the potential effect of urbanisation level (inner-city vs. suburban vs. rural) on annoyance ratings, generalised linear models (GzLMs) were calculated for road and rail with the corresponding *L*_den_ as the covariate with a normal distribution and identify link function. Wave (wave 1 vs. wave 2), mode (online vs. paper–pencil), and region (North vs. East vs. South vs. West) were added for control as potential confounders. GzLMs were chosen as they are rather robust to assumption violations.

To analyse the noise exposure’s (*L*_den_ and *L*_night_) influence on the probability of being highly annoyed (%HA_V_) or highly sleep-disturbed (%HSD), exposure–response analyses were calculated for road traffic noise and railway noise using simple binary logistic regressions using bootstrapping with 5000 bootstrap -samples [36] within the framework of a GzLM. Using bootstrap allowed for the models’ robustness to be estimated. It does not, however, address any potential nonresponse bias (e.g., low SWI). Additional logistic regression analyses using the bootstrap method were performed for noise annoyance and sleep disturbance due to road traffic with level of urbanisation entered as a predictor into the model. Bootstrapping is not frequently applied in noise impact studies; however, it has been utilised by [37], and its application is recommended by [38].

A *p*-value below 0.05 was regarded as statistically significant. IBM SPSS Statistics, version 29.0 and 30.0 (IBM Deutschland GmbH, Ehningen, Germany), and R (version 4.4.2) were used for the statistical analyses.

## 3. Results

### 3.1. Sample Description

In total, 1971 persons participated in the survey; 748 were in wave 1 and 1223 were in wave 2. The gender ratio in the sample was equally distributed, with 50.3% of the participants being male, 49% female, and 0.2% diverse. Four subgroups were formed to derive exposure–response curves for *L*_den_ and *L*_night_ from ≥35 dB for road and rail. Table 1 shows the descriptives of the subgroup in terms of the means, standard deviation, and minimum and maximum values. The mean age was similar in all four subgroups (56.3–56.95), with an age range of 20–94 years. The participants had lived at their current address for an average of 19 years (18.53–18.98). Health status (2.64–2.68) and noise sensitivity were described as average and moderate (2.82–2.86). Residential satisfaction (3.9–4.0) and satisfaction with house/flat (4.09–4.13) were, on average, rated as ‘satisfied’. Socioeconomic status assessed via the Scheuch–Winkler Index (SWI) was categorised into three categories: 54.34% of the sample was in the highest SWI category, 37.75% in the middle SWI, and 5.07% in the lowest SWI category (for 2.84% of the sample, the SWI could not be estimated).

Table 2 and Table 3 show the descriptives of road and rail noise exposure for the subsamples and for each level of urbanisation separately. The mean road traffic *L*_den_ and *L*_night_ are higher (M*_L_*_den_ = 54.4; M*_L_*_night_ = 48.9) compared to the mean rail *L*_den_ (M*_L_*_den_ = 33.2) and *L*_night_ (M*_L_*_night_ = 28.5). When looking at the level of urbanisation, it can be seen that the mean road *L*_den_ and road *L*_night_ are slightly higher in the inner-city and suburban areas compared to rural areas (see Table 3). The opposite is true for rail *L*_den_ and rail *L*_night_, where the mean exposure is higher in rural areas compared to suburban and inner-city areas. For each subgroup, only cases with noise levels ≥ 35 dB for both rail and road *L*_den_ and *L*_night_ were included; the corresponding rail or road levels include levels below 35 dB, i.e., e.g., in the sample 1a only cases with road *L*_den_ ≥ 35 dB are included, but those cases may be exposed to rail *L*_den_ below 35 dB. 

#### Frequency Distribution of Noise Levels *L*_den_ and *L*_night_

**Figure 2:**
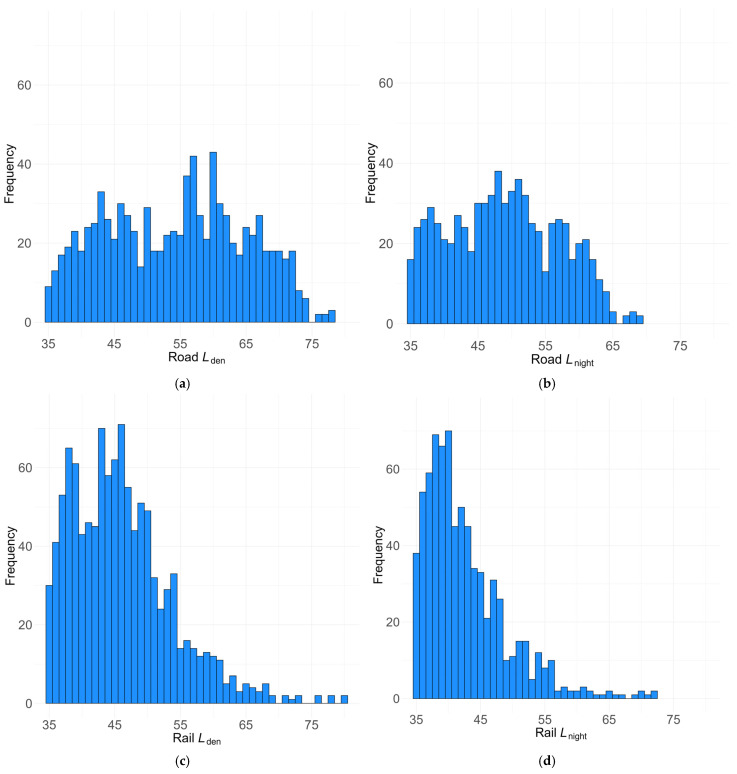
Frequency distribution of day–evening–night values *L*_den_ and night level *L*_night_ for the four subgroups N_Road_ (**a**) road traffic in *L*_den_; (**b**) road traffic at night in *L*_night_; N_Rail_ (**c**) rail traffic in *L*_den_; and (**d**) rail traffic in *L*_nigh_.

Figure 2 illustrates noise exposure in terms of the frequency distribution of noise levels: day–evening–night noise level *L*_den_ and nocturnal noise exposure *L*_night_ of the noise sources road traffic and rail traffic in the four subgroups Road_*L*_den_, Road_*L*_night_, Rail_*L*_den_, and Rail_*L*_night_. Building each subgroup, cases with values ≥ 35 dB were removed from the sample.

The distribution of missing values varies across noise sources. Noise levels range widely, from 0 dB to nearly 80 dB *L*_den_ and 72 dB *L*_night_, depending on the source. Across both sources, a considerable number of respondents were not exposed to specific sources, contributing to low mean levels when exposure levels < 35 dB are not omitted.

Road traffic data are relatively normally distributed across both *L*_den_ and *L*_night_ ranges between 35 dB and 75/65 dB (Figure 1), whereas rail exposure data are more right-skewed distributed for *L*_den_ and *L*_night_.

Table 4 shows the descriptives as well as %HA_v_ and %HSD_v_ for the three levels of urbanisation separately.

### 3.2. Relationships Between Noise Exposure, Non-Acoustical Factors, and Outcome Variables

Correlation analyses were performed to investigate the relationship between annoyance evaluations and sleep disturbance with the different noise exposure levels and other relevant non-acoustic factors. The results are shown in Table A1, Table A2, Table A3 and Table A4 in Appendix A. In the correlation diagrams, colour indicates the direction of the correlation: blue represents a positive correlation, while red denotes a negative correlation. The intensity of the colour corresponds to the strength of the correlation.

**Figure 3:**
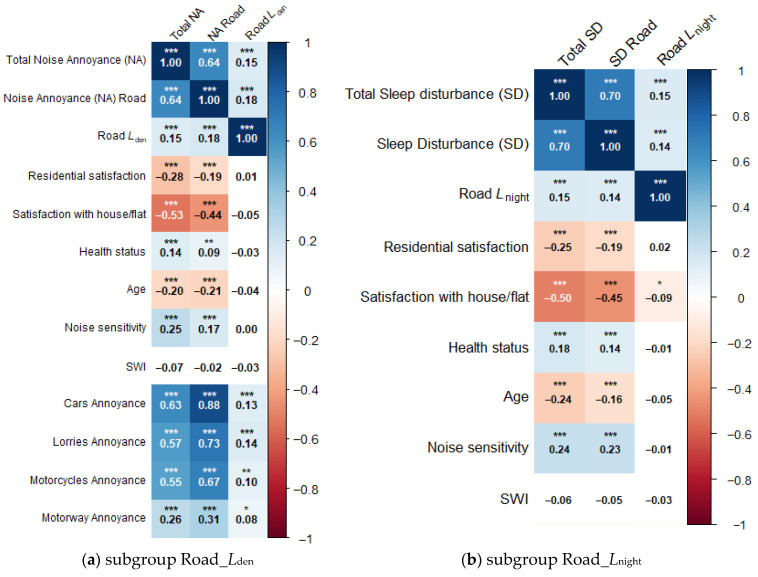
Relationship between road (**a**) noise annoyance and (**b**) sleep disturbance and acoustic parameters (*L*_den_, *L*_night_) and non-acoustic factors and vehicle-specific noise annoyance. TNA = total noise annoyance, NA = noise annoyance, TSD = total sleep disturbance, SD = sleep disturbance, SWI = Scheuch–Winkler Index, *** *p* < 0.001, ** *p* < 0.01, * *p* < 0.05, plain text: not significant.

Pearson’s correlations for the relationship between annoyance (a) and sleep disturbance (b) due to road noise, exposure variables *L*_den_ and *L*_night_, non-acoustical factors, and vehicle-specific annoyance are shown in Figure 3.

The results show that overall perceived noise annoyance is most strongly associated with annoyance from road traffic noise (*r* = 0.64, *p* < 0.001). Among specific sources, road traffic noise annoyance exhibits the highest correlation with annoyance from cars (*r* = 0.88, *p* < 0.001). Strong negative associations are shown between satisfaction with house/flat and total noise annoyance (*r* = −0.53, *p* < 0.001) and road traffic noise annoyance (*r* = −0.44, *p* < 0.001) as well as total sleep disturbance (*r* = −0.50, *p* < 0.001) and sleep disturbance from road traffic noise (*r* = −0.45, *p* < 0.001). Only small correlations are shown between noise levels and outcome variables with *L*_den_/noise annoyance (*r* = 0.18, *p* < 0.001) and *L*_night_/sleep disturbance (r = 0.14; *p* < 0.001). All non-acoustic factors do not, or with a very low effect size (*r* < 0.01), correlate with noise levels. For example, satisfaction with house/flat has a small negative significant association with road *L*_night_ (*r* = −0.09, *p* < 0.05), respectively.

**Figure 4:**
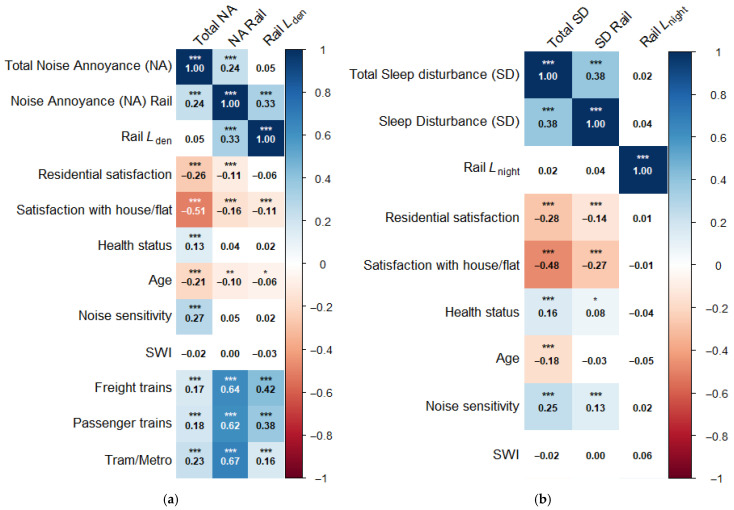
Relationship between rail (**a**) noise annoyance and (**b**) sleep disturbance and acoustic parameters (*L*_den_, *L*_night_) and non-acoustic factors and vehicle-specific noise annoyance. TNA= total noise annoyance, NA = noise annoyance, TSD = total sleep disturbance, SD = sleep disturbance, SWI = Scheuch–Winkler Index, *** *p* < 0.001, ** *p* < 0.01, * *p* < 0.05, plain text: not significant.

Figure 4 illustrates the relationship between railway noise annoyance (a) and sleep disturbance due to railway noise (b) with the respective noise levels and relevant non-acoustic factors. Strong correlations are observed between railway noise annoyance and annoyance from passenger trains (*r* = 0.62, *p* < 0.001), freight trains (*r* = 0.64, *p* < 0.001), and trams/subway trains (*r* = 0.67, *p* < 0.001). The correlation matrix reveals low correlations (*r* < 0.25) between noise levels *L*_night_ and sleep disturbance judgments (*r* = 0.04, *p* < 0.001) but considerable correlation between annoyance and *L*_den_ (*r* = 0.33, *p* < 0.001). Also, residential satisfaction does not significantly correlate with *L*_den_ levels of road and railway noise.

### 3.3. Assessment of Potential Effect of Urbanisation Level

To assess the potential effect of urbanisation level (inner-city vs. suburban vs. rural) on annoyance ratings, GzLMs with a normal distribution and an identify link function were calculated for road and rail with the corresponding *L*_den_ as the covariate. Wave (wave 1 vs. wave 2), mode (online vs. paper–pencil), and region (North vs. East vs. South vs. West) were added for control as potential confounders. The results are depicted in Table 5 and Table A5 and Table A6 in Appendix A.

There is no significant effect of urbanisation level on noise annoyance due to road traffic (Wald χ^2^(2) = 3.673, *p* = 0.159) nor railway traffic (Wald χ^2^(2) = 3.505, *p* = 0.173; see Table A5). For the four different regions in Germany (North, East, South, West), noise annoyance ratings significantly differ regarding road traffic noise (Wald χ^2^(3) = 23.614, *p* < 0.001) and railway noise (Wald χ^2^(3) = 17.549, *p* < 0.001; see Table 5).

### 3.4. Exposure–Response Analyses for %HA and %HSD

The exposure–response functions were calculated using logistic regression analyses with bootstrapping (N_bootstrap_ = 5000) to assess the noise exposure’s impact on the probability of being highly annoyed (%HA_V_) or highly sleep-disturbed (%HSD_V_). Two exposure–response functions were computed for road traffic noise with *L*_den_ and *L*_night_ as predictors and noise annoyance and self-reported sleep disturbance due to road traffic as outcomes, respectively. The same analyses were carried out for railway noise. The curves are presented from 35 dB onwards to minimise potential bias in the exposure–response relationship resulting from discrepancies between the source of the calculated noise levels and the source(s) referenced in the annoyance assessments.

**Figure 5:**
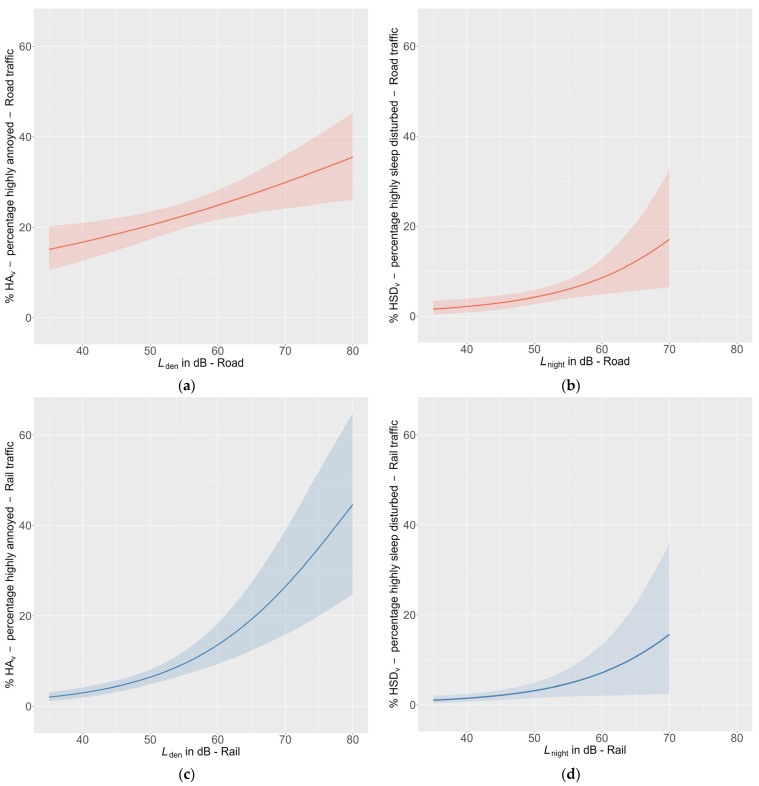
Exposure–response curves with N_bootstrap_ = 5000 for (**a**) the relationship between the day–evening–night level *L*_den_ and %HA_V_ for road traffic noise; (**b**) the relationship between the night level *L*_night_ and %HSD_V_ for road traffic noise; (**c**) the relationship between the day–evening–night level *L*_den_ and %HA_V_ for railway noise; (**d**) the relationship between the night level *L*_night_ and %HSD_V_ for railway noise. The coloured areas around the curves represent the confidence intervals.

The results of the logistic regression analyses can be found in Table 6, Table 7, Table 8 and Table 9. The low bias value (close to 0) indicates that all models are robust to distributions of personal characteristics within the sample. All four logistic regression show a statistically significant influence of the two noise exposures on both outcomes. For instance, exposure to road traffic noise operationalised with *L*_den_ is linked to an increased probability of being highly annoyed by this noise source (B = 0.025, BCI = 0.009–0.043, *p* = 0.001; see Table 6). Further, the probability of being highly sleep-disturbed due to railway noise is significantly predicted by night-time railway noise (*L*_night_; B = 0.0081, BCI = 0.019–0.138, *p* ≤ 0.01; see Table 9). The exposure–response curves are depicted with confidence intervals in Figure 5. At 40 dB *L*_den_ for road traffic noise, approximately 16% are highly annoyed (see Figure 5a) while 3%HA_V_ is reached at 40 dB *L*_den_ for railway (Figure 5c). The exposure–response curve for the %HA_V_ railway is much steeper than that for road traffic, but it starts at a much lower %HA_V_. In addition, both exposure–response curves for sleep disturbance are lower than those for noise annoyance. 3%HSD_V_ is reached at approximately 45 dB *L*_night_ for road traffic and at 49 dB *L*_night_ for railway.

## 4. Discussion

This study investigated the noise annoyance situation in Germany for two traffic noise sources (road and rail), covering four study regions and representing three levels of urbanisation.

Exposure–response curves were calculated based on bootstrap samples for road traffic noise and railway noise for *L*_den_ and *L*_night_. The WHO Environmental Noise Guidelines recommend to not exceed 10% HA and 3% HSD to avoid an increased risk of adverse health effects from noise [4]. The exposure–response curve for road traffic noise level *L*_den_ and road traffic noise annoyance was higher in comparison with the WHO curve derived in a meta-analysis [2], reaching 10% HA by 35 dB *L*_den_ road, whereas 10% HA for road traffic was obtained by 53 dB in the WHO curve. However, this seems to resemble a trend of German road traffic noise as the German curves represented in the meta-analysis exceeded other international studies and are also higher than the resulting average [2]. Furthermore, the vast majority of the original studies included in the WHO meta-analysis for annoyance [2] refer to the definition of HA based on the 11-point numerical ICBEN annoyance scale (HA_N_). The definition of HA_N_ is, with a cut-off of 72% of the annoyance scale for defining HA, stricter, including less people being highly annoyed than according to the HA_V_ definition used in this study. Contradicting results were found in a newer study in Germany using the LIFE cohort where the road exposure–response curves were significantly lower than those obtained in the meta-analysis conducted for the WHO reviews [8]. However, the results of the referenced study are based on data from a single region in the east of Germany, reflecting the noise situation in a region with relatively flat terrain and lower population density. In contrast, the current study was set in regions with varied topography and different overall population density resulting in different traffic volumes and, consequently, a broader range of potential noise exposure scenarios. Discrepancies in the ERR for road traffic noise annoyance compared to other studies might be explained by the underestimation of the respondents’ actual exposure levels. Specifically, modelled noise exposure in some studies may not fully account for contributions from minor roads, as noise mapping under the Environmental Noise Directive (END) is typically limited to major road networks. This exclusion of secondary road network systems may result in potentially underestimating the actual total exposure to major and secondary road networks. In contrast, the LIFE study incorporated estimates of noise exposure from both major and secondary road networks [8], providing a more comprehensive assessment. Assuming that the LIFE study’s noise exposure estimates are more accurate, the consistently lower ERR observed in that study remains notable. The elevated annoyance levels related to road traffic noise compared to other European countries may be attributable to differences in speed regulations, resulting in significantly higher overall exposure and other specific types of noise (e.g., sounds of acceleration). Additionally, expectations regarding disturbance from road traffic noise may influence an individual’s perception of the noise. Perception of noise is further influenced by other non-acoustic factors such as the resources to deal with noise, i.e., when coping mechanisms are insufficient to adapt to noise exposure.

The nocturnal effects of road traffic noise on sleep disturbance were in line with the WHO results as 3% HSD_V_ was reached at *L*_night_ = 45 dB (WHO: 45 dB, [3]). The results for railway noise annoyance were in line with the WHO’s results, reaching 10% HA_V_ at 54 dB *L*_den_; however, the exposure–response curve for the nocturnal effects of railway noise on sleep disturbance was significantly lower in the German study (3% HSD_V_ at 53 dB *L*_night_ in comparison to 44 dB *L*_night_ WHO, [3]). These lower levels of sleep disturbance reported in response to nocturnal railway noise suggest a greater acceptance of railway noise at night, despite the fact that freight train traffic in Germany exceeds the European average. Attitudes towards the noise source can vary. In some areas, people may be more accepting of railway noise; for example, when associated with mobility or the supply of goods and therefore considered non-avoidable traffic. In fact, rail traffic was more positively evaluated compared to road traffic [39]. Additionally, characteristics like noise sensitivity may differ in the present sample compared to the WHO meta-analyses. The results showed that an increase in noise sensitivity was related to an increase in sleep disturbance due to railway noise, but the effects were only small. Another reason could be that bedroom orientation could have buffered the effects of railway noise in this population.

In the present study, a unique feature was the stratified sampling of participants from areas with varying levels of urbanisation. This approach enabled the analysis of how different degrees of urbanisation, ranging from rural environments to inner-city areas, influence the effects of specific noise sources on annoyance responses.

Notably, the impact of road traffic noise did not vary across levels of urbanisation, even after controlling for exposure levels (*L*_den_). Road traffic noise annoyance was similarly pronounced in the inner-city area compared to the suburban area and the rural area, indicating that the derived exposure–response curves apply across area types. In contrast, other studies found differences in noise annoyance between area types. Ref. [9] found noise annoyance due to road traffic to be significantly more pronounced in the urban areas compared to other area types. The authors argued that in their study, the expectation for it to be quiet was lower in suburban and urban areas compared to rural/remote areas. However, these findings were solely based on the assessment of the impact without considering individual exposure assuming potential confounding between noise levels and the degree of urbanisation. Therefore, differences in annoyance ratings between the urbanised areas may be attributable to varying noise exposure. Similar, the GEDA study classified the living context of their sample in metropolitan, urban, and rural districts, showing greater road noise annoyance with increasing density of the living district [11], also not controlling for actual exposure at residents’ houses. It must be stated that the classification of areas with different levels of urbanisation differed in comparison with the current study. Systematically including participants from rural areas, suburban, and inner-city locations, this study was able to depict a representative geographic picture while considering different urbanisation levels. There are several reasons to take into consideration differences in noise annoyance due to urbanisation, even when not supported by the data. Rural areas are often assumed to have lower background noise levels, so residents may have higher expectations of quietness. In inner-city areas, people might expect and accept higher noise levels as part of urban living, leading to a certain degree of habituation or acceptance. Conversely, given the lower expectations of quietness in urban areas, annoyance ratings might be expected to be lower as well. Suburban areas may have a more mixed soundscape—neither as quiet as rural areas nor as consistently noisy as inner cities. People in these areas might be less habituated to constant noise but still experience significant exposure. However, even moderate levels of road traffic noise can be perceived as intrusive and annoying because they contrast sharply with the typically quieter environment. This could be shown for aircraft noise annoyance being higher with increasing emergence, i.e., for aircraft noise levels in otherwise quieter areas compared to the same aircraft noise level in otherwise “louder” areas [13]. In contrast, ref. [39] showed no systematic trend in the *L*_eq_ range for traffic noise (road, rail, air) for different urbanisation levels. Greenery is often associated with more rural areas. Indeed, there is evidence that greenery has a beneficial effect on annoyance levels when controlled for noise exposure [40,41]. However, ref. [40] showed that this effect of greenery is not moderated by the degree of urbanisation, supporting the assumption that there may be distinct features of certain areas that influence noise annoyance rather than the classification of area types regarding urbanisation. Unfortunately, we do not have data on greenery. For future research, we recommend assessing both greenery and urbanisation level to generate a better understanding of the potential effects. Future studies should also include the assessment of participants’ expectations regarding the acoustic environment at home, in particular quietness, as well as information about background noise.

Correlation analyses show no or very low effect size correlations (*r* < 0.01) between all non-acoustic factors and noise levels, hinting to the assumption that these factors are less likely direct effects of noise exposure. Further, the fact that satisfaction with one’s house/flat is slightly but statistically significantly correlated with road and railway noise exposure (*L*_den_ and *L*_night_) may indicate effects of vibration and secondary noise occurring in parallel to noise exposure—in particular railway noise exposure—or it may indicate that people are unsatisfied with the sound insulation indoors, not protecting them enough against the traffic noise. Considering that outdoor spaces like gardens, balconies, or community spaces are inherently part of houses/flats, noise exposure could affect the perceived liveability in these spaces. However, these are only assumptions that cannot be verified with the existing study data but may be worthwhile to be studied in future research.

This sampling design with two waves, one in spring and one in autumn, allowed for a more nuanced understanding of how contextual factors interact with environmental noise exposures to impact perceived annoyance. No seasonal effect was found for the two waves (spring vs. autumn). In contrast, other studies such as the SIRENE study demonstrated seasonal effects on annoyance rates; in particular, greater annoyance was reported in autumn compared to spring [32]. Further, considering the nested structure of the data (e.g., four regions), multilevel or hierarchical modelling could be applied for exploratory purposes in future analyses, provided that the data fulfil the requirements for these statistical methods.

The study design applied in this study followed a range of criteria for the selection of study areas that were suitable to map the noise annoyance situation, including considering areas with varying levels of urbanisation whilst considering the distribution of noise exposure. This aim to represent the noise annoyance situation in Germany considering the level of urbanisation was achieved. However, exposure–response curves are less reliably derived from this design, as the simple random selection of participants based on the degree of urbanisation rather than the stratified random sampling with noise exposure as stratum results in the insufficient representation of individuals across all exposure levels; that is, in the higher exposure categories. For robust exposure–response functions, this design is only suitable when investigating very common noise sources with a relatively uniform distribution of exposure levels across a broad range of occurring levels. However, although further stratification by exposure levels would have exceeded the initial scope and resources of this study, sample distribution covered the main range of noise levels.

Additionally, the quality of noise exposure assessment relies heavily on the availability of noise data from local authorities, which was constrained for certain noise sources in some study areas. Therefore, future studies aiming to comprehensively assess the noise situation (i.e., exposure–response curves) should adopt sampling strategies that ensure sufficient numbers of participants across the full range of noise exposure levels.

Newer studies indicate that noise maps differ in terms of data formats and exposure assessment influencing the estimations of exposed people [42]. In this study, the noise exposure was modelled using EU noise mapping data, which only consider major roads. Only cases from 35 dB *L*_den_ were considered in this study. Cases with lower noise levels were excluded from the analysis, as a mismatch can occur between the source of the noise levels (e.g., from road A) and the source of the annoyance judgement (rating of noise from road B), i.e., annoyance judgement refers to other sources of exposure than the calculated levels [43]. Furthermore, in addition to noise exposure from roads included in the noise maps as specified by the European Noise Directive, annoyance judgments may also relate to noise levels from residential roads not covered by these maps. The setting of 35 dB was determined because the source level should be above the background noise level, including the consideration of exposure assessment uncertainty. According to the NORAH study, the lowest background noise level in the night was estimated to be below 30 dB [44]. Acknowledging the uncertainty estimation, we decided to start from 35 dB. Considering the available resources, more precise exposure data could be attained by using more complete input data.

The estimation of noise effects on noise annoyance followed the approach recommended by the ICBEN committee (40% on the annoyance and sleep disturbance scale, i.e., cut-off for HA_V_ = 60%), slightly differing from the effect estimation of 28% (cut-off for HA_N_ = 72%) used in the vast majority of original studies included in the WHO meta-analysis on environmental noise annoyance [2]. Both are consistent with ISO15666. However, a direct comparison between the exposure–response curves of this study with the WHO curves has to be interpreted with caution.

Another limitation is the response rate, which was quiet low in the first wave. Firstly, a substantial proportion of letters could not be delivered to the intended recipients, raising concerns about the accuracy and completeness or timeliness of the address data supplied by the address service company. Secondly, the participation in the first wave of the survey was limited to the online mode, preventing the participation of certain groups due to a lack of internet connection, affinity, or age. Thirdly, switching from the invitation by post to online participation could represent an increased effort and implicitly reduce motivation to participate.

A comparison of the socioeconomic characteristics of the study sample with national averages indicates overall representativeness in terms of age [45], gender [46], and education [47], with some deviations. Gender distribution closely matches the national average, with differences of no more than ±2%. The sample includes a lower proportion of individuals under 40 years old and a higher proportion of those aged 40 to 80 compared to the 2019 national average. Individuals with an income below EUR 3000 are underrepresented relative to the 2019 national average [48]. Participants with higher educational attainment are overrepresented compared to the 2019 national average. Lower socioeconomic status (SES) is associated with higher levels of noise exposure in residential environments [49]. Moreover, the likelihood of being annoyed by noise from road or rail traffic appears to be elevated among individuals with low SES compared to those with higher SES [11]. Therefore, future studies should aim to achieve a balanced distribution in their sample in terms of socioeconomic status to minimise potential effects. However, using the bootstrap method in the present study, which applies a high number of resampling data, derives exposure–response relationships that are more robust to the effects of individual characteristics such as age, gender, or education than standard exposure–response procedures.

## 5. Conclusions

The current study assessed the noise annoyance situation in Germany across four larger regions stratified by three different levels of urbanisation, using a cross-sectional design with 1971 participants. This study evaluated both noise levels and the effects of two main noise sources: road traffic and railway noise. The results show that road traffic noise emerges as the most significant problem, with exposure–response curves surpassing the international average, indicating that lower levels of road traffic noise cause greater annoyance in Germany compared to other countries. Railway noise annoyance displayed a steeper slope at higher exposure levels, suggesting that individuals in Germany may be more sensitive to increases in railway noise compared to other regions. Choosing the derived ERFs over the WHO curves would have a significant effect on the results from the health risk assessment. Overall, these findings highlight the varying effects of different noise sources, suggesting that more targeted interventions are needed to address the most problematic noise sources in specific contexts.

In this study, we used the most recent available German supraregional data for the exposure response analysis on road and railway noise annoyance. As it is known that environmental noise annoyance may vary over time (e.g., [50,51]), it is recommended that future research periodically surveys the exposure–response relationship in order to allow for up-to-date urban and infrastructural planning and noise management, in line with [52].

## Figures and Tables

**Table 1:** Descriptives of the total sample and the subgroups for road and rail.

Variable	N	M	SD	Min	Max
Sample 1a: Road_*L*_den_ ≥ 35 dB (N = 900)					
Age	885	56.9	13.9	21	92
Period of residence	879	18.5	12.8	0	81.2
Health status	892	2.7	0.9	1	5
Noise sensitivity	887	2.9	1.0	1	5
Residential satisfaction	888	4.0	0.9	1	5
Satisfaction with house/flat	877	4.1	0.8	1	5
Sample 1b: Road_*L*_night_ ≥ 35 dB (N = 730)					
Age	715	56.8	14.0	21	92
Period of residence	712	18.6	13.0	0	81.2
Health status	724	2.6	0.9	1	5
Noise sensitivity	719	2.9	1.0	1	5
Residential satisfaction	719	4.0	0.9	1	5
Satisfaction with house/flat	712	4.2	0.8	1	5
Sample 2a: Rail_*L*_den_ ≥ 35 dB (N = 1099)					
Age	1080	56.9	14.2	20	94
Period of residence	1079	19.0	13.0	0	81.2
Health status	1093	2.7	0.9	1	5
Noise sensitivity	1086	2.8	1.0	1	5
Residential satisfaction	1080	4.0	0.9	1	5
Satisfaction with house/flat	1072	4.1	0.8	1	5
Sample 2b: Rail_*L*_night_ ≥ 35 dB (N = 753)					
Age	737	56.3	14.1	20	92
Period of residence	735	18.9	13.2	0	81.2
Health status	748	2.7	0.9	1	5
Noise sensitivity	742	2.8	1.0	1	5
Residential satisfaction	740	3.9	0.9	1	5
Satisfaction with house/flat	733	4.1	0.9	1	5

Note. N = number, M = mean, SD = standard deviation, min = minimum, max = maximum.

**Table 2:** Descriptive statistics of noise exposure (*L*_den_ and *L*_night_) of road and rail traffic in the four subgroups.

Noise Exposure to Noise Sources	N	Min	Max	Median	M	SD
Sample 1a: Road_*L*_den_ ≥ 35 dB (N = 900)						
Road	900	35.0	78.0	55.4	54.4	10.5
Sample 1b: Road_*L*_night_ ≥ 35 dB (N = 730)						
Road	730	35.0	69.0	48.8	48.9	8.1
Sample 2a: Rail_*L*_den_ ≥ 35 dB (N = 1099)						
Rail	1099	35.0	79.8	45.1	46.1	7.7
Sample 2b: Rail_*L*_night_ ≥ 35 dB (N = 753)						
Rail	753	35.0	72.1	40.9	42.7	6.4

Note: N = sample size; Min = minimum; Max = maximum; M = mean; SD = standard deviation.

**Table 3:** Descriptive statistics of noise exposure (*L*_den_ and *L*_night_) to main noise sources for different levels of urbanisation (inner-city, suburban, rural).

Noise Exposure to Noise Sources	N	Min	Max	Median	M	SD
Sample 1a: Road_*L*_den_ ≥ 35 dB (N = 900)						
Inner-city	511	35	69.0	50.1	49.8	8.1
Suburban	183	35.1	64.4	46.7	47.1	7.5
Rural	36	35.1	63.4	44.6	45.4	7.6
Sample 1b: Road_*L*_night_ ≥ 35 dB (N = 730)						
Inner-city	639	35	78.0	56.3	55.1	10.9
Suburban	218	35.4	74.1	53.5	53.3	9.2
Rural	43	35.8	71.6	47.9	50.0	8.8
Sample 2a: Rail_*L*_den_ ≥ 35 dB (N = 1099)						
Inner-city	467	35.0	72.1	40.0	41.7	6.3
Suburban	175	35.2	68.6	43.3	44.0	6.1
Rural	111	35.1	66.9	44.0	44.8	6.8
Sample 2b: Rail_*L*_night_ ≥ 35 dB (N = 753)						
Inner-city	467	35.0	72.1	40.0	41.7	6.3
Suburban	175	35.2	68.6	43.3	44.0	6.1
Rural	111	35.1	66.9	44.0	44.8	6.8

Note: N = sample size; Min = minimum; Max = maximum; M = mean; SD = standard deviation.

**Table 4:** Descriptives for annoyance and sleep disturbance and %HA_v_ and %HSD_v_ per urbanisation level.

	Inner-City				Suburban				Rural			
**Noise** **Annoyance**	**N**	**M**	**SD**	**%HA_V_**	**N**	**M**	**SD**	**%HA_V_**	**N**	**M**	**SD**	**%HA_V_**
Road ^1^	615	2.6	1.2	25.0	209	2.2	1.1	14.8	41	2.6	1.4	26.8
Rail ^2^	675	1.4	0.9	6.1	209	1.4	0.8	3.8	132	1.8	1.0	6.8
**Sleep** **Disturbance**	**N**	**M**	**SD**	**%HSD_V_**	**N**	**M**	**SD**	**%HSD_V_**	**N**	**M**	**SD**	**%HSD_V_**
Road ^3^	491	1.7	1.0	4.5	165	1.5	0.8	4.8	33	1.9	1.1	6.1
Rail ^4^	442	1.4	0.7	1.6	159	1.3	0.8	3.8	105	1.4	0.7	1.9

^1^ Road *L*_den_ ≥ 35 dB (N = 900); ^2^ Rail *L*_den_ ≥ 35 dB (N = 1099); ^3^ Road *L*_night_ ≥ 35 dB (N = 730); ^4^ Rail *L*_night_ ≥ 35 dB (N = 753).

**Table 5:** Tests of model effects.

	Source	Wald Chi-Square	df	*p*
1 road *	(Intercept)	0.001	1	0.970
	Wave	3.402	1	0.065
	Mode	0.164	1	0.685
	Region	23.614	3	0.000
	Level of urbanisation	3.673	2	0.159
	*L*_den_	14.677	1	<0.001
2 rail **	(Intercept)	3.474	1	0.062
	Wave	0.003	1	0.957
	Mode	0.033	1	0.856
	Region	17.549	3	0.001
	Level of urbanisation	3.505	2	0.173
	*L*_den_	31.329	1	<0.001

df = degrees of freedom; *p* = significance level; * road *L*_den_ ≥ 35 dB (N = 900); ** rail *L*_den_ ≥ 35 dB (N = 1099).

**Table 6:** Logistic regression analysis for *L*_den_ and noise annoyance (HA_V_) due to road traffic noise.

Parameter	B	SE	95% BCI Lower	95% BCI Upper	*p*	Bias
(Constant)	−2.632	0.493	−3.603	−1.704	0.000	−0.009
Road traffic *L*_den_	0.025	0.009	0.009	0.043	0.001	0.000

Note: B = bootstrap regression coefficient, SE = standard error, 95%-BCI = 95% bootstrap confidence interval, *p* = significance level.

**Table 7:** Logistic regression analysis for *L*_night_ and sleep disturbance (HSD_V_) due to road traffic noise.

Parameter	B	SE	95% BCI Lower	95% BCI Upper	*p*	Bias
(Constant)	−6.859	1.462	−9.989	−4.214	0.000	−0.126
Road traffic *L*_night_	0.074	0.027	0.023	0.130	0.002	0.002

Note: B = bootstrap regression coefficient, SE = standard error, 95%-BCI = 95% bootstrap confidence interval, *p* = significance level.

**Table 8:** Logistic regression analysis for *L*_den_ and noise annoyance (HA_V_) due to railway noise.

Parameter	B	SE	95% BCI Lower	95% BCI Upper	*p*	Bias
(Constant)	−6.788	0.728	−8.284	−5.414	0.000	−0.028
Railway *L*_den_	0.082	0.014	0.055	0.110	0.000	0.000

Note: B = bootstrap regression coefficient, SE = standard error, 95%-BCI = 95% bootstrap confidence interval, *p* = significance level.

**Table 9:** Logistic regression analysis for *L*_night_ and sleep disturbance (HSD_V_) due to railway noise.

Parameter	B	SE	95% BCI Lower	95% BCI Upper	*p*	Bias
(Constant)	−7.535	1.435	−10.390	−4.726	0.000	−0.041
Railway *L*_night_	0.081	0.030	0.019	0.138	0.004	−0.001

Note: B = bootstrap regression coefficient, SE = standard error, 95%-BCI = 95% bootstrap confidence interval, *p* = significance level.

## Data Availability

Dataset available on request from the authors.

## Appendix A

**Table A1:** Pearson’s correlations between total noise annoyance, noise annoyance due to road noise, and road *L*_den_, as well as with noise annoyance due to their single components, exposure variables, and non-acoustic factors.

		Total Noise Annoyance	Noise Annoyance Road	Road *L*_den_
Total noise annoyance	*r*	1.00	0.64	0.15
	*p*	0.00	0.00	0.00
	*N*	894	860	894
Noise Noise annoyance road	*r*	0.64	1.00	0.18
	*p*	0.00	0.00	0.00
	*N*	860	865	865
Road *L_den_*	*r*	0.15	0.18	1.00
	*p*	0.00	0.00	0.00
	*N*	894	865	900
Residential satisfaction	*r*	−0.28	−0.19	0.01
	*p*	0.00	0.00	0.85
	*N*	892	862	896
Satisfaction with house/flat	*r*	−0.53	−0.44	−0.05
	*p*	0.00	0.00	0.18
	*N*	884	856	888
Health status	*r*	0.14	0.09	−0.03
	*p*	0.00	0.01	0.42
	*N*	886	858	892
Age	*r*	−0.20	−0.21	−0.04
	*p*	0.00	0.00	0.26
	*N*	880	851	885
Noise sensitivity	*r*	0.25	0.17	0.00
	*p*	0.00	0.00	0.92
	*N*	881	853	887
SWI	*r*	−0.07	−0.02	−0.03
	*p*	0.05	0.48	0.36
	*N*	863	834	869
Cars annoyance	*r*	0.63	0.88	0.13
	*p*	0.00	0.00	0.00
	*N*	834	814	837
Lorries annoyance	*r*	0.57	0.73	0.14
	*p*	0.00	0.00	0.00
	*N*	820	804	823
Motorcycles annoyance	*r*	0.55	0.67	0.10
	*p*	0.00	0.00	0.00
	*N*	833	814	837
Motorway annoyance	*r*	0.26	0.31	0.08
	*p*	0.00	0.00	0.02
	*N*	857	834	860

*r* = Pearson’s correlation coefficient, *p* = significance level, *N* = number of participants.

**Table A2:** Pearson’s correlations between total sleep disturbance, sleep disturbance due to road noise, road *L*_night_, and exposure variables and non-acoustic factors.

		Total Sleep Disturbance	Sleep Disturbance Road	Road *L_night_*
Total sleep disturbance	*r*	1.00	0.70	0.15
	*p*	0.00	0.00	0.00
	*N*	695	683	695
Sleep disturbance road	*r*	0.70	1.00	0.14
	*p*	0.00	0.00	0.00
	*N*	683	689	689
Road *L_night_*	*r*	0.15	0.14	1.00
	*p*	0.00	0.00	0.00
	*N*	695	689	730
Residential satisfaction	*r*	−0.25	−0.19	0.02
	*p*	0.00	0.00	0.55
	*N*	691	685	726
Satisfaction with house/flat	*r*	−0.50	−0.45	−0.09
	*p*	0.00	0.00	0.01
	*N*	684	680	719
Health status	*r*	0.18	0.14	−0.01
	*p*	0.00	0.00	0.88
	*N*	690	684	724
Age	*r*	−0.24	−0.16	−0.05
	*p*	0.00	0.00	0.17
	*N*	681	675	715
Noise sensitivity	*r*	0.24	0.23	−0.01
	*p*	0.00	0.00	0.78
	*N*	686	680	719
SWI	*r*	−0.06	−0.05	−0.03
	*p*	0.14	0.19	0.37
	*N*	667	660	701

*r* = Pearson’s correlation coefficient, *p* = significance level, *N* = number of participants.

**Table A3:** Pearson’s correlations between total noise annoyance, noise annoyance due to rail noise, and rail *L*_den_, as well as with noise annoyance due to their single components, exposure variables, and non-acoustic factors.

		Total Noise Annoyance	Noise Annoyance Rail	Rail *L*_den_
Total noise annoyance	*r*	1.00	0.24	0.05
	*p*	0.00	0.00	0.11
	*N*	1092	1011	1092
Noise annoyance rail	*r*	0.24	1.00	0.33
	*p*	0.00	0.00	0.00
	*N*	1011	1016	1016
Rail *L_den_*	*r*	0.05	0.33	1.00
	*p*	0.11	0.00	0.00
	*N*	1092	1016	1099
Residential satisfaction	*r*	−0.26	−0.11	−0.06
	*p*	0.00	0.00	0.06
	*N*	1089	1012	1095
Satisfaction with house/flat	*r*	−0.51	−0.16	−0.11
	*p*	0.00	0.00	0.00
	*N*	1074	1003	1080
Health status	*r*	0.13	0.04	0.02
	*p*	0.00	0.17	0.53
	*N*	1086	1012	1093
Age	*r*	−0.21	−0.10	−0.06
	*p*	0.00	0.00	0.04
	*N*	1073	1000	1080
Noise sensitivity	*r*	0.27	0.05	0.02
	*p*	0.00	0.09	0.57
	*N*	1079	1005	1086
SWI	*r*	−0.02	0.00	−0.03
	*p*	0.56	0.94	0.33
	*N*	1053	983	1060
Freight trains	*r*	0.17	0.64	0.42
	*p*	0.00	0.00	0.00
	*N*	989	962	994
Passenger trains	*r*	0.18	0.62	0.38
	*p*	0.00	0.00	0.00
	*N*	984	960	989
Tram/metro	*r*	0.23	0.67	0.16
	*p*	0.00	0.00	0.00
	*N*	1001	965	1006

*r* = Pearson’s correlation coefficient, *p* = significance level, *N* = number of participants.

**Table A4:** Pearson’s correlations between total sleep disturbance, sleep disturbance due to railway noise, rail *L*_night_, and exposure variables and non-acoustic factors.

		Total Sleep Disturbance	Sleep Disturbance Rail	Rail *L_night_*
Total sleep disturbance	*r*	1.00	0.38	0.02
	*p*	0.00	0.00	0.55
	*N*	718	699	718
Sleep disturbance rail	*r*	0.38	1.00	0.04
	*p*	0.00	0.00	0.26
	*N*	699	706	706
Rail *L_night_*	*r*	0.02	0.04	1.00
	*p*	0.55	0.26	0.00
	*N*	718	706	753
Residential satisfaction	*r*	−0.28	−0.14	0.01
	*p*	0.00	0.00	0.82
	*N*	714	702	749
Satisfaction with house/flat	*r*	−0.48	−0.27	−0.01
	*p*	0.00	0.00	0.77
	*N*	706	695	740
Health status	*r*	0.16	0.08	−0.04
	*p*	0.00	0.04	0.31
	*N*	715	702	748
Age	*r*	−0.18	−0.03	−0.05
	*p*	0.00	0.47	0.15
	*N*	703	691	737
Noise sensitivity	*r*	0.25	0.13	0.02
	*p*	0.00	0.00	0.56
	*N*	709	696	742
SWI	*r*	−0.02	0.00	0.06
	*p*	0.65	0.99	0.11
	*N*	698	686	731

*r* = Pearson’s correlation coefficient, *p* = significance level, *N* = number of participants.

**Table A5:** Pairwise comparisons for road traffic noise—GzLM results.

(I) Group 1	(J) Group 2	Mean Difference (I-J)	Std.-Error	*p*	95% CI Lower	95% CI Upper
Wave 1	Wave 2	0.079	0.043	0.065	−0.005	0.164
Wave 2	Wave 1	−0.079	0.043	0.065	−0.164	0.164
Online	Paper–pencil	−0.017	0.042	0.685	−0.099	0.065
Paper–pencil	Online	0.017	0.042	0.685	−0.065	0.065
East	West	0.227	0.175	0.196	−0.117	0.571
	North	0.059	0.179	0.743	−0.293	0.410
	South	0.076	0.173	0.662	−0.265	0.416
West	East	−0.168	0.046	<0.001	−0.258	−0.078
	North	−0.151	0.042	<0.001	−0.233	−0.069
	South	0.017	0.057	0.763	−0.095	0.129
North	East	−0.227	0.175	0.196	−0.571	0.571
	West	−0.059	0.179	0.743	−0.410	0,410
	South	−0.076	0.173	0.662	−0.416	0.416
South	East	0.168	0.046	<0.001	0.078	−0.078
	West	0.151	0.042	<0.001	0.069	−0.069
	North	−0.017	0.057	0.763	−0.129	0.129
Inner-city	Suburban	0.065	0.034	0.057	−0.002	0.131
	Rural	0.003	0.069	0.965	−0.133	0.139
suburban	Inner-city	−0.062	0.073	0.400	−0.205	0.082
	Rural	−0.065	0.034	0.057	−0.131	0.131
Rural	Inner-city	−0.003	0.069	0.965	−0.139	0.139
	Suburban	0.062	0.073	0.400	−0.082	0.082

df = degrees of freedom, *p* = significance level, CI = confidence interval.

**Table A6:** Pairwise comparisons for rail noise—GzLM results.

(I) Group 1	(J) Group 2	Mean Difference (I-J)	Std.-Error	*p*	95% CI Lower	95% CI Upper
Wave 1	Wave 2	−0.001	0.023	0.957	−0.046	0.044
Wave 2	Wave 1	0.001	0.023	0.957	−0.044	0.044
Online	Paper–pencil	0.004	0.023	0.856	−0.041	0.049
Paper–pencil	Online	−0.004	0.023	0.856	−0.049	0.049
East	West	0.055	0.034	0.105	−0.011	0.121
	North	0.096	0.035	0.007	0.026	0.165
	South	0.111	0.034	0.001	0.043	0.178
West	East	0.041	0.018	0.026	0.005	0.076
	North	0.056	0.018	0.002	0.021	0.091
	South	0.015	0.020	0.457	−0.024	0.054
North	East	−0.055	0.034	0.105	−0.121	0.121
	West	−0.096	0.035	0.007	−0.165	0.165
	South	−0.111	0.034	0.001	−0.178	0.178
South	East	−0.041	0.018	0.026	−0.076	0.076
	West	−0.056	0.018	0.002	−0.091	0.091
	North	−0.015	0.02	0.457	−0.054	0.054
Inner-city	Suburban	0.034	0.019	0.067	−0.002	0.071
	Rural	0.017	0.023	0.451	−0.028	0.062
suburban	Inner-city	−0.017	0.026	0.518	−0.068	0.034
	Rural	−0.034	0.019	0.067	−0.071	0.071
Rural	Inner-city	−0.017	0.023	0.451	−0.062	0.062
	Suburban	0.017	0.026	0.518	−0.034	0.034

df = degrees of freedom, *p* = significance level, CI = confidence interval.
